# PC-1/PrLZ confers resistance to rapamycin in prostate cancer cells through increased 4E-BP1 stability

**DOI:** 10.18632/oncotarget.3931

**Published:** 2015-05-11

**Authors:** Lan Yu, Zeng-Fu Shang, Jian Wang, Hongtao Wang, Fang Huang, Zhe Zhang, Ying Wang, Jianguang Zhou, Shanhu Li

**Affiliations:** ^1^ Laboratory of Medical Molecular Biology, Beijing Institute of Biotechnology, Beijing 100850, PR China; ^2^ School of Radiation Medicine and Protection, Medical College of Soochow University, Collaborative Innovation Center of Radiation Medicine of Jiangsu Higher Education Institutions, Suzhou, Jiangsu 215123, PR China; ^3^ State Key Laboratory of Experimental Hematology Institute of Hematology and Blood Diseases Hospital, Chinese Academy of Medical Sciences & Peking Union Medical College, Tianjin 300200, PR China

**Keywords:** PC-1/PrLZ, PCa, rapamycin-resistance, mTOR pathway, 4E-BP1

## Abstract

An important strategy for improving advanced PCa treatment is targeted therapies combined with chemotherapy. *PC-1*, a *prostate Leucine Zipper* gene (PrLZ), is specifically expressed in prostate tissue as an androgen-induced gene and is up-regulated in advanced PCa. Recent work confirmed that PC-1 expression promotes PCa growth and androgen-independent progression. However, how this occurs and whether this can be used as a biomarker is uncertain. Here, we report that PC-1 overexpression confers PCa cells resistance to rapamycin treatment by antagonizing rapamycin-induced cytostasis and autophagy (rapamycin-sensitivity was observed in PC-1-deficient (shPC-1) C4-2 cells). Analysis of the mTOR pathway in PCa cells with PC-1 overexpressed and depressed revealed that eukaryotic initiation factor 4E-binding protein 1(4E-BP1) was highly regulated by PC-1. Immunohistochemistry assays indicated that 4E-BP1 up-regulation correlates with increased PC-1 expression in human prostate tumors and in PCa cells. Furthermore, PC-1 interacts directly with 4E-BP1 and stabilizes 4E-BP1 protein via inhibition of its ubiquitination and proteasomal degradation. Thus, PC-1 is a novel regulator of 4E-BP1 and our work suggests a potential mechanism through which PC-1 enhances PCa cell survival and malignant progression and increases chemoresistance. Thus, the PC-1-4E-BP1 interaction may represent a therapeutic target for treating advanced PCa.

## INTRODUCTION

Prostate cancer (PCa) is the most commonly diagnosed cancer and second leading cause of cancer-related deaths in men in Europe and the US [1]. Despite improvements in early screening and treatment, PCa is diagnosed in 240,000 men and causes 30,000 deaths per year in the US [2]. Most patients die from advanced PCa, which is still difficult to treat [3]. Understanding molecular events involved in PCa progression will enable us to experiment with novel therapies. PTEN, a tumor suppressor protein, is often mutated or deleted in advanced PCa, activating the PI3K/Akt/mammalian target of rapamycin (mTOR) pathway which is associated with PCa development and progression [4]. Therefore, this pathway may be a molecular target for PCa treatment.

4E-BP1, eukaryotic initiation factor 4E (eIF4E) binding protein 1, is an mTORC1 substrate that dimerizes with eIF4E, blocking initiation complex formation. When it is phosphorylated by mTORC1, eIF4E is released and cap-dependent translation begins [5]. Most recent work revealed that loss of 4E-BP1 contributes to epithelial-mesenchymal transition (EMT) and cancer cells migration and invasion through promoting cap-dependent translation [6]. However, several evidences supported the idea that 4E-BP1 is recognized as a funnel factor and highly relevant molecular marker of malignant potential [7]. Most advanced breast cancers overexpress 4E-BP1 as well [7–9] total and phosphorylated 4E-BP1 (p-4E-BP1) which is highly expressed in high-grade prostatic intraepithelial neoplasia (HGPIN) may help identify patients at a high risk for tumor development [7]. Braunstein's group revealed that 4E-BP1 activated internal ribosome entry site (IRES)-mediated translation initiation, through which 4E-BP1 facilitates breast cancer angiogenesis and hypoxic responses in animal models [8]. 4E-BP1 is also overexpressed in other human tumors, including prostate, head and neck, colorectal, endometrial, and some gastrointestinal cancers [8, 10–13]. However, how the 4E-BP1 signaling pathway and relative regulation of 4E-BP1 function in cancer development is uncertain. PC-1/PrLZ, a member of the tumor protein D52 (TPD52) family, is induced by androgens and is expressed in prostate tissues. *PC-1* gene expression is low in androgen-dependent, nonmetastatic LNCaP PCa cells, and is up-regulated in androgen-independent, osseous metastatic and LNCaP lineage-related C4-2 cells [14]. Our team and Li's group previously reported that PC-1 expression is prevalently up-regulated in advanced PCa tissues [15, 16], and this promotes PCa cell androgen-dependent and -independent growth [17]. Thus, PC-1 possesses characteristics of oncogenesis. Wang and co-workers [18] reported that PC-1 interacts with 14-3-3 proteins which may be related to the biological function of PC-1. However, the clinical value of PC-1 and how it functions along with its downstream effectors have not been fully elucidated. Here, we show that PC-1 confers PCa cell resistance to the mTOR kinase inhibitor rapamycin. PC-1 overexpression is associated with increased 4E-BP1 expression in human prostate tumors and PC-1 interacts directly with 4E-BP1 to stabilize 4E-BP1 protein via inhibiting ubiquitination and proteasomal degradation. PC-1 overexpression antagonizes rapamycin-induced cell cycle arrest and autophagy, so PC-1 may be a novel molecular therapeutic target for PCa.

## RESULTS

### PC-1 expression confers PCa cells resistance to rapamycin

**Figure 1:**
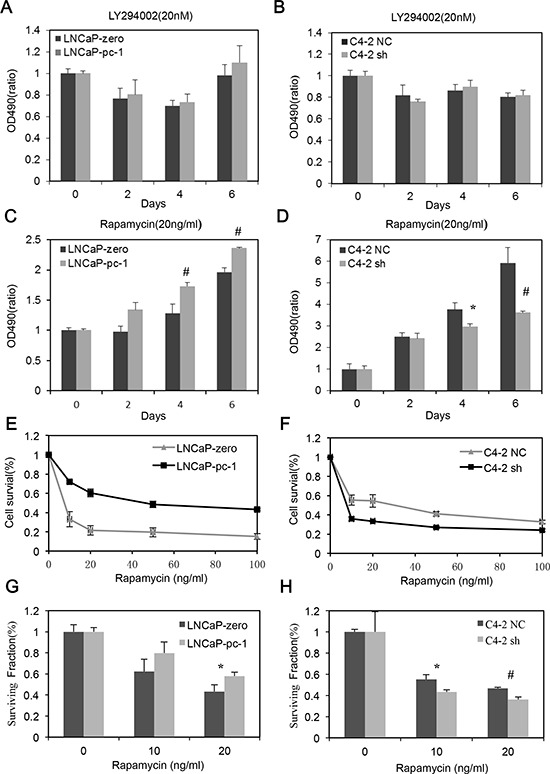
PC-1 expression confers LNCaP and C4-2 cell resistance to rapamycin but not LY294002 **A, B, C** and **D.** cell growth curve analysis. LNCaP and C4-2 subline over- or under-expressing PC-1 were seeded in RPMI 1640 with 8% FBS and treated the next day with 20 nM LY294002 or 20 ng/ml rapamycin. After two days, cells were counted with an MTT assay. **E and F.** normalized cell growth inhibition (Y axis) for the LNCaP and C4-2 subline exposed to increasing concentrations of rapamycin (X axis). Absorbance values are normalized to control. **G and H.** colony-formation assays. LNCaP and LNCaP-PC-1 cells or C4-2 NC and C4-2 sh cells were seeded onto plates, and after 15 days of treatment with/without rapamycin, cells were stained with crystal violet and colonies were counted. (#*p* < 0.01, *p < 0.05 as compared with control cells).

The PI3K/AKT/mTOR pathway has a prominent role in the progression of PCa and is a target therapy of advanced PCa [19]. Therefore, we examined PC-1 expression with respect to PCa cell sensitivity to the PI3K inhibitor LY294002 or to the mTOR inhibitor rapamycin. PC-1 status did not affect chemosensitivity to LY294002 in PCa cells (Fig. 1A and 1B). However, PC-1 expression dramatically increased PCa cell resistance to rapamycin (Fig. 1C and 1D). With rapamycin (10–100 ng/ml) PC-1 overexpression significantly decreased LNCaP cell sensitivity to rapamycin (Fig. 1E) and PC-1 silencing by RNA interference (RNAi) strongly increased C4-2 cell sensitivity to rapamycin (Fig. 1F). Furthermore, we measured PCa cell survival after rapamycin treatment using a colony-formation assay. The results were consistent to the previous observation; PCa cells which expressed PC-1 were more resistant to rapamysin (Fig. 1G and 1H). Thus, altering PC-1 expression in PCa cells alters sensitivity to rapamycin but not to LY294002.

### PC-1 upregulates eukaryotic initiation factor 4E-binding protein 1 (4E-BP1) expression

**Figure 2:**
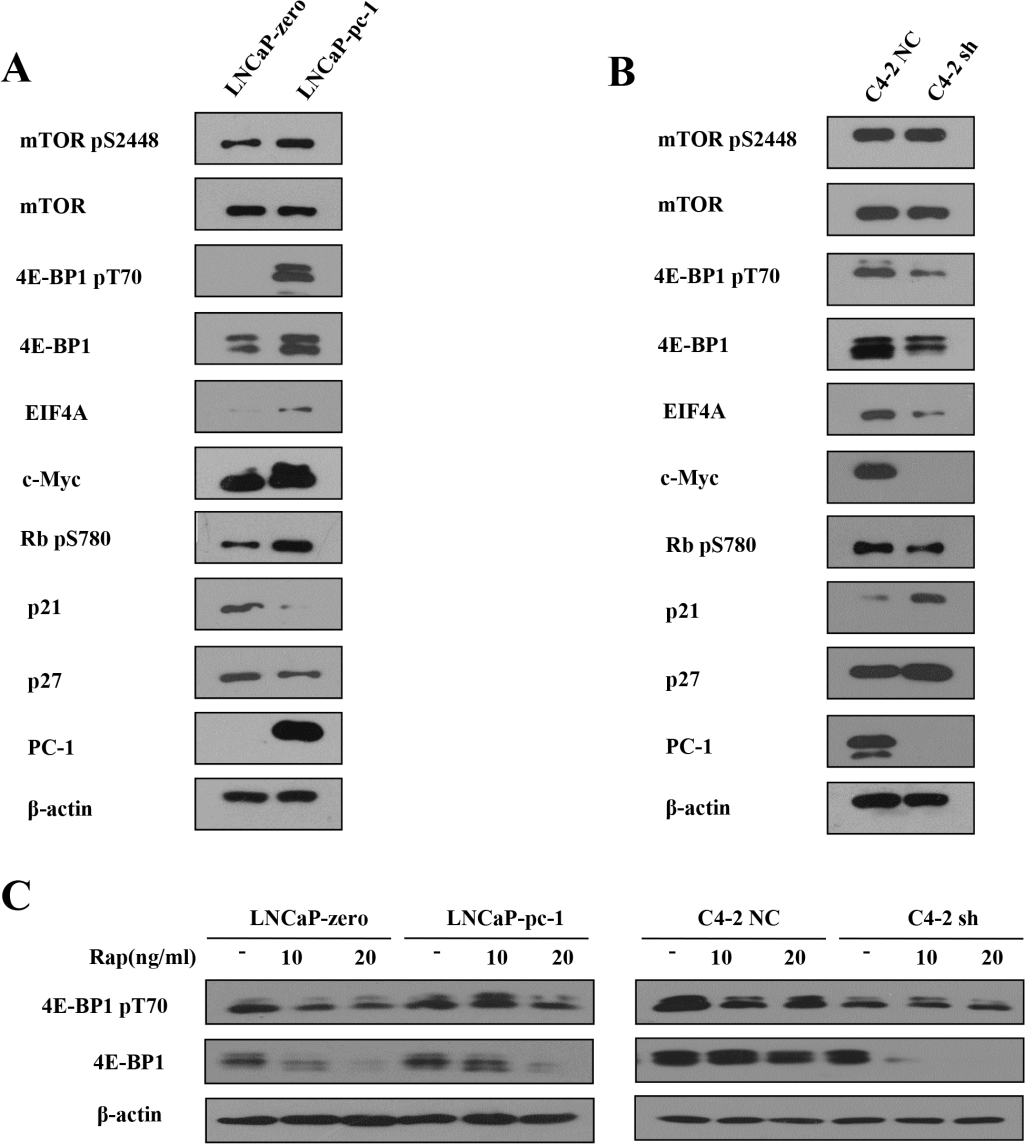
PC-1 expression increased 4E-BP1 protein expression **A and B.** Expression and phosphorylation of mTOR and mTOR effector proteins in an LNCaP and C4-2 subline as described in Figure 1. Cells were grown to log phase in normal culture and proteins were extracted. **C.** Protein expression and phosphorylation of 4E-BP1 in LNCaP and C4-2 subline after treatment with different concentrations of rapamycin. Cells were grown to log phase in normal culture and incubated for 48 h in serum-starved medium and then transferred to normal medium for 24 h with/without rapamycin.

To determine the molecular mechanisms of PC-1 on rapamycin resistance in PCa cells, we first investigated the effect of PC-1 on the mTOR signaling pathway in LNCaP and C4-2 cells. PC-1 overexpression significantly increased total and phosphorylated 4E-BP1, whereas total and phosphorylated mTOR were not changed (Fig. 2A). Conversely, PC-1 knockdown decreased total and phosphorylated 4E-BP1 (Fig. 2B). 4E-BP1 may be a funnel factor for essential oncogenic capability in tumor cells [7], we therefore analyzed whether PC-1 affected expression of growth-related genes. c-Myc is a well characterized target of 4E-BP1, and as a transcription factor, c-Myc can directly activate transcription of three subunits of eIF4F (eIF4E, eIF4AI, and eIF4GI) [20]. Here, we show that expression of c-Myc and eIF4A correlated with PC-1 expression. As shown in Fig. 2A, stable transfection of pcDNA3.1 B(−)-PC-1 into LNCaP cells enhanced phosphorylation of RB at Ser^780^ and decreased p21 and p27 expression compared with control. Phosphorylation of RB at Ser^780^ was reduced and p21 and p27 expression increased in PC-1 depleted C4-2 sh cells compared with control (Fig. 2B). Thus, PC-1 expression stimulates PCa growth even after rapamycin treatment. Also, PC-1 increased expression and phosphorylation of 4E-BP1 in LNCaP cells treated with 10 or 20 ng/ml rapamycin, but 4E-BP1 expression was lower in C4-2 sh cells compared to control C4-2 NC cells after treatment with 10 or 20 ng/ml rapamycin (Fig. 2C).

### PC-1 upregulation is associated with increased 4E-BP1 expression in human prostate cells and prostate tumors

**Figure 3:**
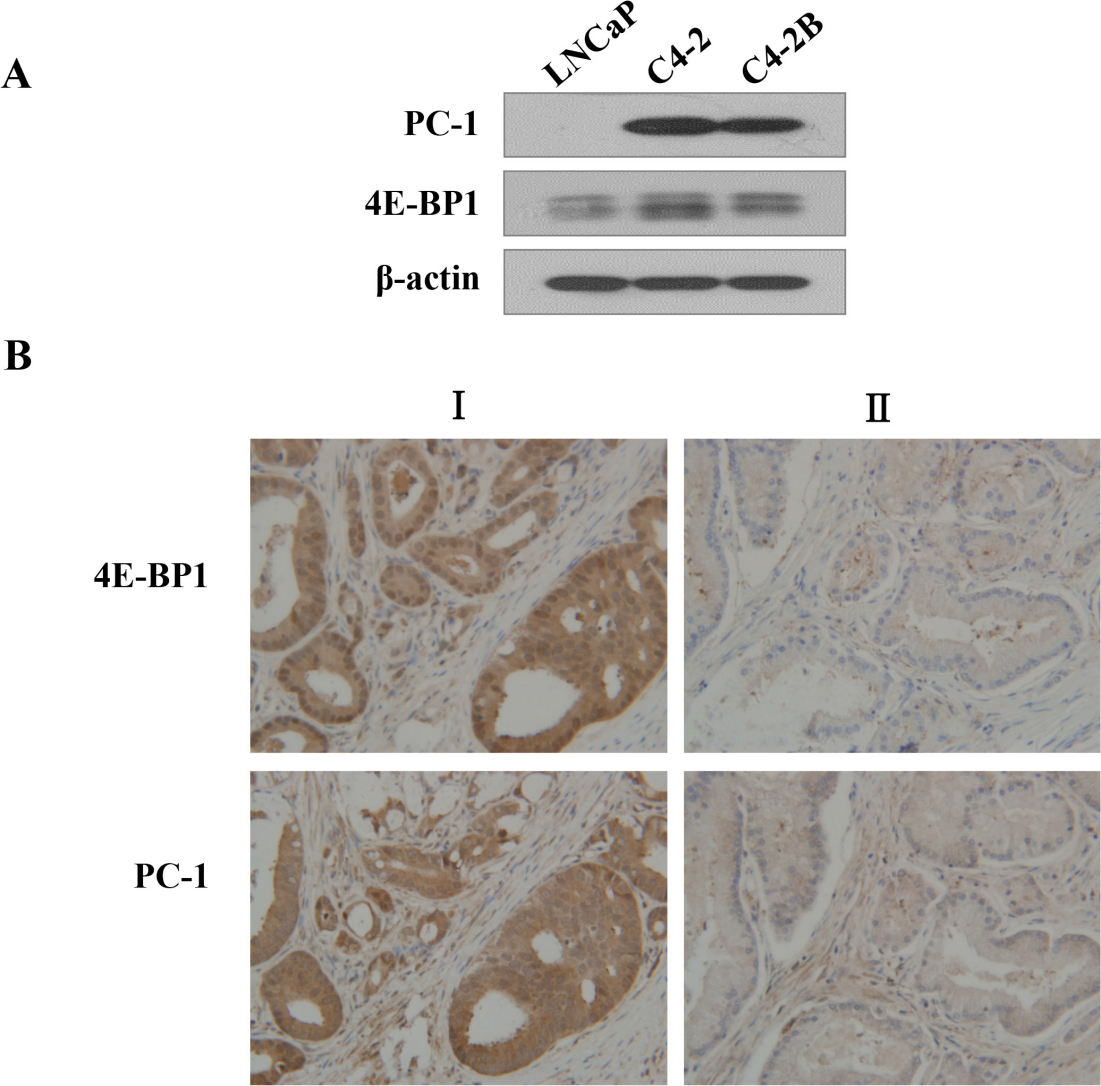
4E-BP1 expression is consistently correlated with PC-1 expression in human PCas and cells **A.** Expression of 4E-BP1 and PC-1 in PCa cell lines by Western blot. **B.** immunohistochemical studies of human prostate adenocarcinomas. Human PCa tissues were immunostained with antibodies against 4E-BP1 and PC-1. Representative tissue sections from tumor I, a high PC-1 expression carcinoma containing high 4E-BP1, mainly in the cytoplasm of the prostate epithelia. Representative sections from tumor II, PC-1 and 4E-BP1 expression is low.

PC-1 expression is up-regulated in advanced PCa tissue [15]. To define the correlation between PC-1 and 4E-BP1, we measured PC-1 expression and 4E-BP1 in PCa cell lines and tissue arrays of 40 prostate tumors via Western blot and immunohistochemistry, respectively. We observed a relationship between PC-1 expression and 4E-BP1 (Fig. 3; Table 1). In PCa cells, 4E-BP1 protein was higher in androgen-independent and osseous metastatic C4-2 and C4-2B cells compared to LNCaP cells (Fig. 3A), similar to PC-1 expression data in an LNCaP/C4-2 PCa progression model [15]. PC-1 is highly expressed in clinical samples with greater 4E-BP1 (representative case I in Fig. 3B), consistently, in tumors for which PC-1 and 4E-BP1 expression is low (representative case II in Fig. 3B). Thus, up-regulation of 4E-BP1 is correlated with increased PC-1 expression in human prostate tumors suggesting that up-regulation of PC-1 might be key to 4E-BP1-mediated prostate tumorigenesis.

**Table 1:** PC-1 and 4E-BP1 expression is correlated in prostate tumor tissues

	PC-1 (high)	PC-1 (low)	Total
4E-BP1 (high)	24 (60%)	2 (5%)	26 (65%)
4E-BP1 (low)	9 (22.5%)	5 (12.5%)	14 (35%)
Total	33 (82.5%)	7 (17.5%)	40 (100%)

Values are number (percentage), *p* = 0.03531

### PC-1 interacts directly with 4E-BP1

**Figure 4:**
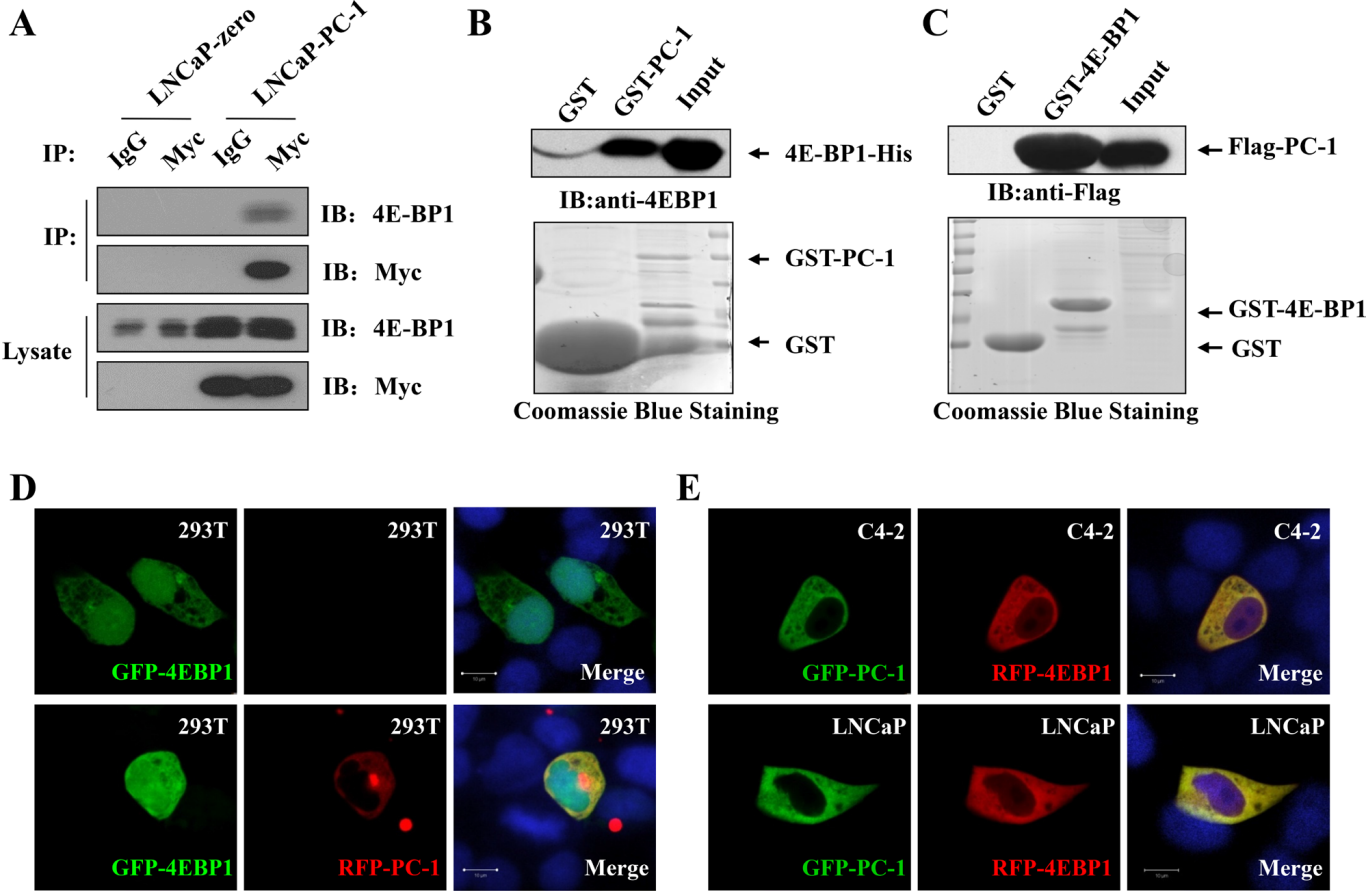
PC-1 directly interacts with 4E-BP1 **A.** LNCaP-zero and LNCaP-pc-1 cells were harvested and lysates used to immunoprecipitate PC-1 using rabbit anti-myc antibodies or rabbit IgG as control. The immunoprecipitates were resolved by SDS-PAGE and transferred onto nitrocellulose membranes which were immunoblotted with anti-4E-BP1 and anti-PC-1 antibody. **B and C.** GST pull-down assays. B. Both input and pull-down samples were subjected to immunoblotting with anti-His antibody. Coommassie staining of the purified GST-PC-1 or GST proteins is shown at the bottom. C. 293T cells transfected with pCMV-2B-PC-1 and the cell lysates were incubated with GST-4E-BP1 or GST. Bound proteins were analyzed by immunoblotting using anti-Flag antibody. Equal loading of lysates and GST-4E-BP1 or GST indicated by Coommassie staining. **D and E.** Colocalization analyses of PC-1 and 4E-BP1. D. 293T cells were grown on glass coverslip and transfected with GFP-4E-BP1 (row 1) or cotransfected with GFP-4E-BP1 and RFP-PC-1 (row 2), cells were fixed after 48 h and analyzed using cofocal microscopy. Nuclei were stained with DAPI. E. LNCaP and C4-2 cells were cotransfected with GFP-PC-1 and RFP-4E-BP1 and analyzed as D.

To confirm the role of PC-1 in the regulation of 4E-BP1, we examined whether PC-1 interacted directly with 4E-BP1 using glutathione *S*-transferase (GST) pull-down assays with recombinant GST-PC-1 and His-4E-BP1 (Fig. 4B) or GST-4E-BP1 and cell lysates transfected with Flag-PC-1 (Fig. 4C). Specific interaction of PC-1 with 4E-BP1 *in vitro* was observed. Furthermore, we performed co-immunoprecipitation assays with LNCaP-zero and LNCaP-pc-1 cell lysates. Endogenous 4E-BP1 could be co-precipitated with Myc-PC-1 (Fig. 4A). Immunofluorescent staining revealed that exogenous 4E-BP1 localized predominantly to the nuclei and less to the cytoplasm. When 293T, LNCaP and C4-2 cells were cotransfected with GFP-PC-1 and RFP-4E-BP1, PC-1 colocalized to the cytoplasm with 4E-BP1 (Fig. 4D and 4E). Thus, PC-1-4E-BP1 interaction was observed in the both the tagged over-expression experiment as well as the endogenous experiment.

### PC-1 affects stability and ubiquitination of 4E-BP1 protein

**Figure 5:**
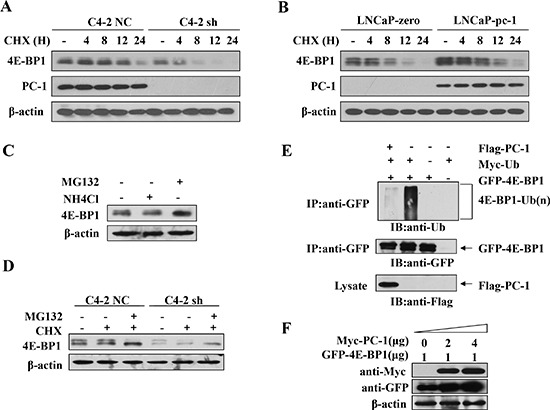
PC-1 regulates 4E-BP1 stability **A.** 4E-BP1 protein is degraded rapidly after PC-1 silencing in C4-2 cell. Cells were treated with cycloheximide (CHX) and were harvested at 0, 4, 8, 12 and 24 h after CHX treatment. **B.** 4E-BP1 protein is degraded slowly in the presence of PC-1 in LNCaP cells. Cells were treated with CHX and harvested at 0, 4, 8, 12, and 24 h after CHX treatment. 4E-BP1 without CHX treatment was a control. **C.** Proteasome inhibitor MG132 but not lysomoal protease inhibitor NH_4_Cl recovered 4E-BP1 degradation in C4-2 cells. 1640 medium was used as a control. **D.** MG132 recovered 4E-BP1 downregulation in C4-2 sh cells. **E.** Enhanced 4E-BP1 protein expression in the presence of increasing PC-1 *in vitro*. GFP-4E-BP1 was co-transfected with increasing Myc-PC-1 in LNCaP cells. **F.** PC-1 decreased ubiquitination of 4E-BP1. 2.0 μg Myc-ubiquitin, 1.0 μg Flag-PC-1 and GFP-4E-BP1 were single or co-transfected. Cells were harvested 18 h after MG132 (10 μM) treatment. 4E-BP1 was immunoprecipitated using anti-GFP antibody. CoIP product was analyzed by immunoblotting with anti-ubiquitin antibody, Equal loading of CoIP product immunoblotted with anti-GFP and anti-Flag antibodies.

To elucidate how PC-1 regulates 4E-BP1 protein, we investigated whether PC-1 regulated stability of 4E-BP1 protein. We measured 4E-BP1 half-time by measuring 4E-BP1 protein alteration after blocking protein synthesis with cycloheximide (CHX). We compared 4E-BP1 protein between PC-1-proficient or -deficient cells and controls at different time points after CHX treatment. 4E-BP1 appeared relatively stable in C4-2 NC cells compared with C4-2 sh cells (Fig. 5A). Also, 4E-BP1 appeared more stable in LNCaP-pc-1 cells compared to control cells. At 12 and 24 h after CHX treatment, 4E-BP1 protein decreased more slowly in LNCaP-pc-1 cells compared with control LNCaP cells (Fig. 5B). Thus, PC-1 maintains 4E-BP1 protein stability. We then evaluated whether PC-1-mediated 4E-BP1 protein stability relies on an ubiquitination-mediated proteosomal pathway. We measured 4E-BP1 protein in C4-2 cells in the presence of MG132, a specific inhibitor of the proteosome or NH_4_Cl, which could inhibit lysosomal protease. MG132 blocked 4E-BP1 degradation, whereas NH_4_Cl did not (Fig. 5C). MG132 also could restore 4E-BP1 expression in PC-1-deficient C4-2 sh cells (Fig. 5D). Next, we transfected 4E-BP1 together with Ub, PC-1 expression plasmid and measured 4E-BP1 ubiquitination. As shown in Fig. 5E, signals of polyubiquintinated 4E-BP1 in PC-1 expressed cells were weaker than in cells without PC-1. Furthermore, PC-1 dose-dependently enhanced 4E-BP1 expression in 293T cells (Fig. 5F). These data suggest that PC-1 promotes 4E-BP1 stability and impedes ubiquitination of 4E-BP1.

### PC-1 expression alters rapamycin-induced cytostasis and autophagy

**Figure 6:**
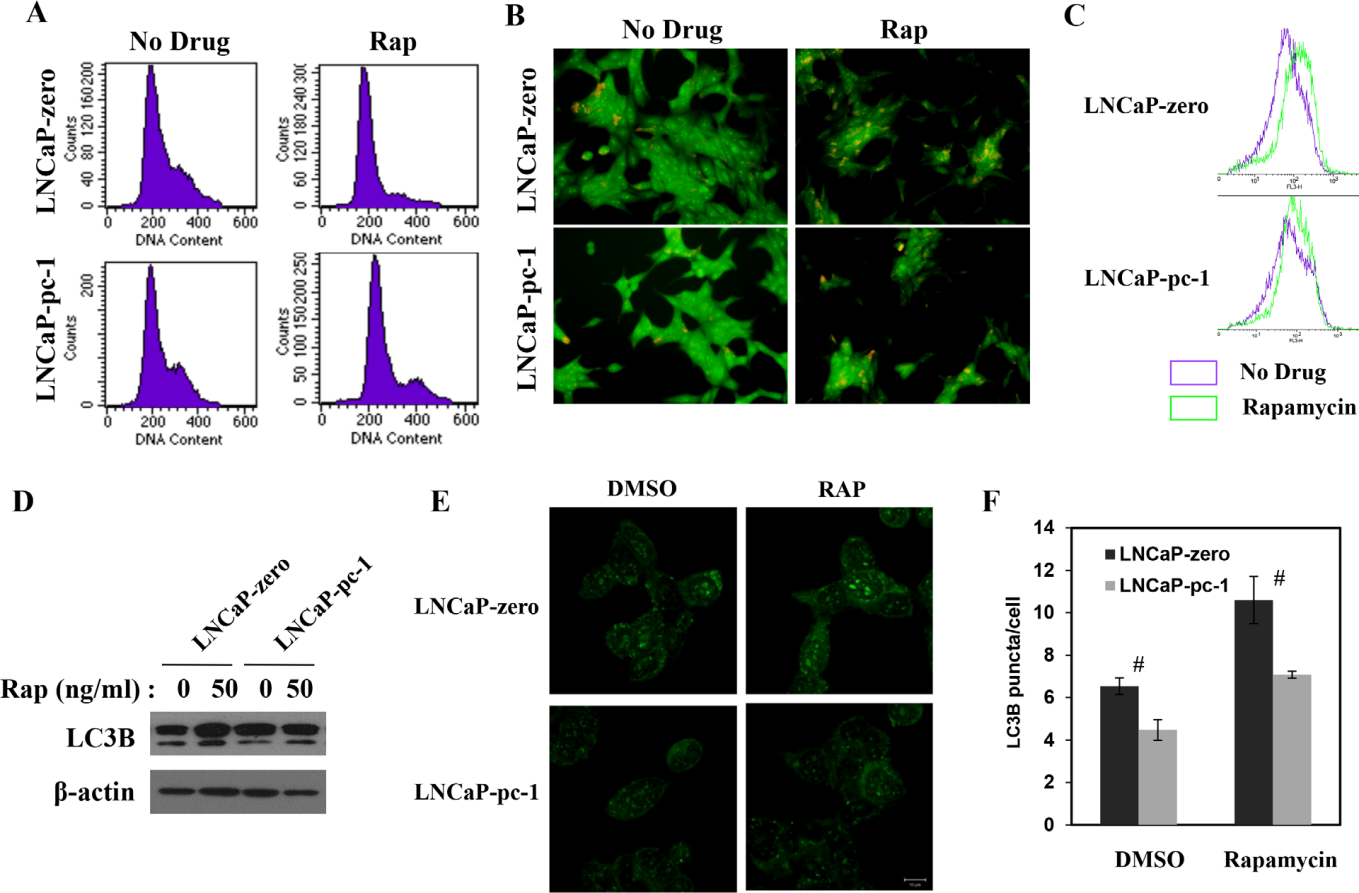
PC-1 alters rapamycin-induced cytostasis and autophagy **A.** LNCaP and LNCaP-pc-1 cells were fixed 24 h after treatment with/without rapamycin (Rap) and cell cycle analysis by flow cytometry. **B.** Cells were grown in the presence/absence of 20 ng/ml rapamycin for 24 h and fluorescent images of acridine orange-positive cells are shown. Cells stained orange underwent autophagy. **C.** Autophagy analyzed with flow cytometry (See “B” for treatment). **D.** LC3B protein was measured via immunoblotting for autophagy after exposure for 24 h to 50 ng/ml rapamycin. **E.** Immunofluorescence (IF) showing the puncta of LC3 in LNCaP-zero and LNCaP-pc-1 cells in presence or absence rapamycin (50 ng/ml). **F.** Mean number of LC3 puncta in rapamycin treated or untreated LNCaP-zero and LNCap-pc-1 cells (*n* = 3; error bars, s.e.m.). (#*p* < 0.01 as compared with control cells).

Inhibiting 4E-BP1 with rapamycin should increase severe cytostasis [21, 22], so we investigated whether PC-1 expression affected rapamycin-induced cytostasis in PCa cells. We measured cell cycle distribution of LNCaP/LNCaP-pc-1 or C4-2 NC/C4-2 sh cells after rapamycin treatment with flow cytometry. In LNCaP cells, rapamycin treatment arrested cells in the G_0_/G_1_ phase. Expression of PC-1 abrogates rapamycin-induced G_0_/G_1_ phase arrest (Fig. 6A). In contrast, PC-1 silencing increases rapamycin-induced G_0_/G_1_ phase arrest (data not shown). Except for the role in cell cycle regulation, 4E-BP1 plays an important role in inhibiting autophagy [23, 24]. Inhibition of 4E-BP1 could sensitize PCa cells to rapamycin-induced autophagy [25, 26], so we studied autophagy in LNCaP and LNCaP-pc-1 cells using acridine orange staining, flow cytometry, and LC3B Western blot and immunofluorescence after rapamycin treatment (Fig. 6B, 6C, 6D, 6E and 6F) as described in Balakumaran's study [26]. LNCaP and LNCaP-pc-1 cells were treated with rapamycin (20 ng/ml), and 24 h were stained with acridine orange for 20 min. Analysis of autophagy by fluorescent microscope and flow cytometry revealed that PC-1 expression limited rapamycin-induced autophagy (Fig. 6B and 6C). We next measured LC3B protein by Western blot. PC-1 expression abrogated increased LC3B protein induced by rapamycin (Fig. 6D). We also performed the immunostaining with LC3B antibody (Fig. 6E and 6F) and the results were consistent with Fig. 6D. Therefore, PC-1 expression can partially overcome rapamycin-induced cytostasis and autophagy.

## DISCUSSION

Recently we showed that PC-1 contributes to PCa androgen-dependent and -independent progression and malignant phenotypes, and its expression stimulates the Akt/protein kinase B signaling pathway in PCa cells [27]. Also, we studied biological functions of the *PC-1* gene in PCa progression and downstream effectors of this gene.

Androgen receptor (AR) activation, triggered by androgens, is key to prostate development and PCa progression. Therefore, surgery or chemotherapeutic-mediated androgen-deprivation therapies (ADT) are effective initial approaches for treatment of prostate tumor progression. However, PCa will progress to aggressive cancers that are resistant to castration therapy and can be lethal [28]. Thus, advanced prostate tumors remain a significant therapy challenge. The PI3K/AKT/mTOR pathway is activated in advanced PCa because of a mutation or deletion of PTEN [29, 30]. Leontieva et al. reported that the inhibition of mTOR by rapamycin led to cellular pseudo-hypoxia state through HIF-1 dependent signal pathway even in normoxia condition, indicating its crucial role in cellular metabolism regulation [31]. Thus, mTOR is an attractive therapeutic target, and some mTOR inhibitors have been studied in PCa cells [29]. Preclinical studies suggest that rapamycin analogues CCI-779 and RAD001 inhibit proliferation of human PCa cell lines, especially PTEN-deficient LNCaP and PC-3 cells [25, 32]. In this study, growth curve and colony-formation assays revealed that rapamycin inhibited the growth of LNCaP cells and a lineage-related C4-2 subline [33, 34], and that PC-1 overexpression antagonizes LNCaP cell sensitivity to rapamycin, whereas PC-1-depletion in C4-2 cells had increased rapamycin sensitivity. Thus, silencing PC-1 and rapamycin treatment significantly inhibit PCa cell proliferation.

To investigate the mechanism behind the function of PC-1 in rapamycin resistance, we examined the effect of PC-1 expression on the mTOR signaling pathway. Western blot revealed that PC-1 overexpression increases total and phosphorylated 4E-BP1. In contrast, silencing PC-1 expression in C4-2 correspondingly decreases total and phosphorylated 4E-BP1 in Thr^70^. However, total mTOR and pmTOR were not obviously changed. Furthermore, PC-1 regulates expression of growth-related genes regulated by the mTOR signaling pathway, such as p21, p27, and phosphorylation of RB at Ser^780^. These results revealed that PC-1 antagonizes growth inhibition and cytostasis induced by rapamycin via regulating cell survival- and proliferation-related genes. 4E-BP1 knockdown increased baseline and rapamycin-induced autophagy [26]. Thus, PC-1 could overcome rapamycin-induced autophagy and cell cycle arrest via maintenance of 4E-BP1 protein stability. These observations point to a direct relationship between PC-1 and 4E-BP1. Demidenko and coworkers revealed that inhibition of mTOR by rapamycin prevented permanent loss of proliferation ability of cell and therefore converted cells from senescence status into quiescence [35]. It will be interesting to test whether PC-1-4E-BP1 signal pathway contributes to cells proliferation potential preservation.

4E-BP1, the eIF4E-binding protein 1, interacts with eukaryotic translation initiation factor eIF4E and suppresses the formation of the eukaryotic translation initiation factor 4F (eIF4F) complex, which regulates cap-dependent translation initiation in mammalian cells [8]. mTOR phosphorylates 4E-BP1 and blocks its association with eIF4E when cells are stimulated with insulin, growth factors and other types of extracellular stimuli [36–38]. mTOR inhibitor rapamycin may inhibit the phosphorylation of 4E-BP1 and prevent cap-dependent translation initiation. Here, we found that PC-1 antagonizes rapamycin-induced depression of the 4E-BP signal pathway. Due to its role in cap-dependent translation initiation, 4E-BP1 is thought of as an inhibitory factor of cell or tumor survival and proliferation [6, 39–41]. However, no evidence confirms that 4E-BP1 is lost or decreased in tumors. In contrast, studies suggest that 4E-BP1 expression is elevated in the majority of human cancers, including advanced breast, prostate, head and neck, colorectal, endometrial, and some gastrointestinal cancers [8, 10–13]. We also identified that 4E-BP1 is overexpressed in human prostate carcinomas via a tissue microarray analysis. Using the same method, PC-1 and 4E-BP1 expression was correlated in prostate tumor tissues (Table 1). In response to cellular stress, cap-dependent translation is shut-down and global translation is severely comprised to save energy needed for stress [42]. At the same time, a subset of mRNA, which is required for a stress response, is still efficiently or even more translated through an alternative mode of translation initiation, IRES-mediated translation. Many proteins translated in an IRES-dependent manner play important roles in cell survival, proliferation and angiogenesis [43, 44]. Evidence suggests that in addition to its role in cap-dependent translation initiation, 4E-BP1 may also promote IRES-dependent translation initiation under certain conditions, such as hypoxia [8]. Cap-dependent translation is inhibited during mitosis progression and some mitotic regulators are translated via IRES-dependent translation [45–47]. Previous data by our group and others confirmed that 4E-BP1 could regulate mitosis progression [48, 49]. Therefore, 4E-BP1 might coordinate cap-dependent and -independent mRNA translation initiation under some stress conditions and mitotic progression, which promotes tumor progression and development. Then, future studies can determine whether PC-1 participates in translation initiation via a 4E-BP1-dependent pathway.

**Figure 7:**
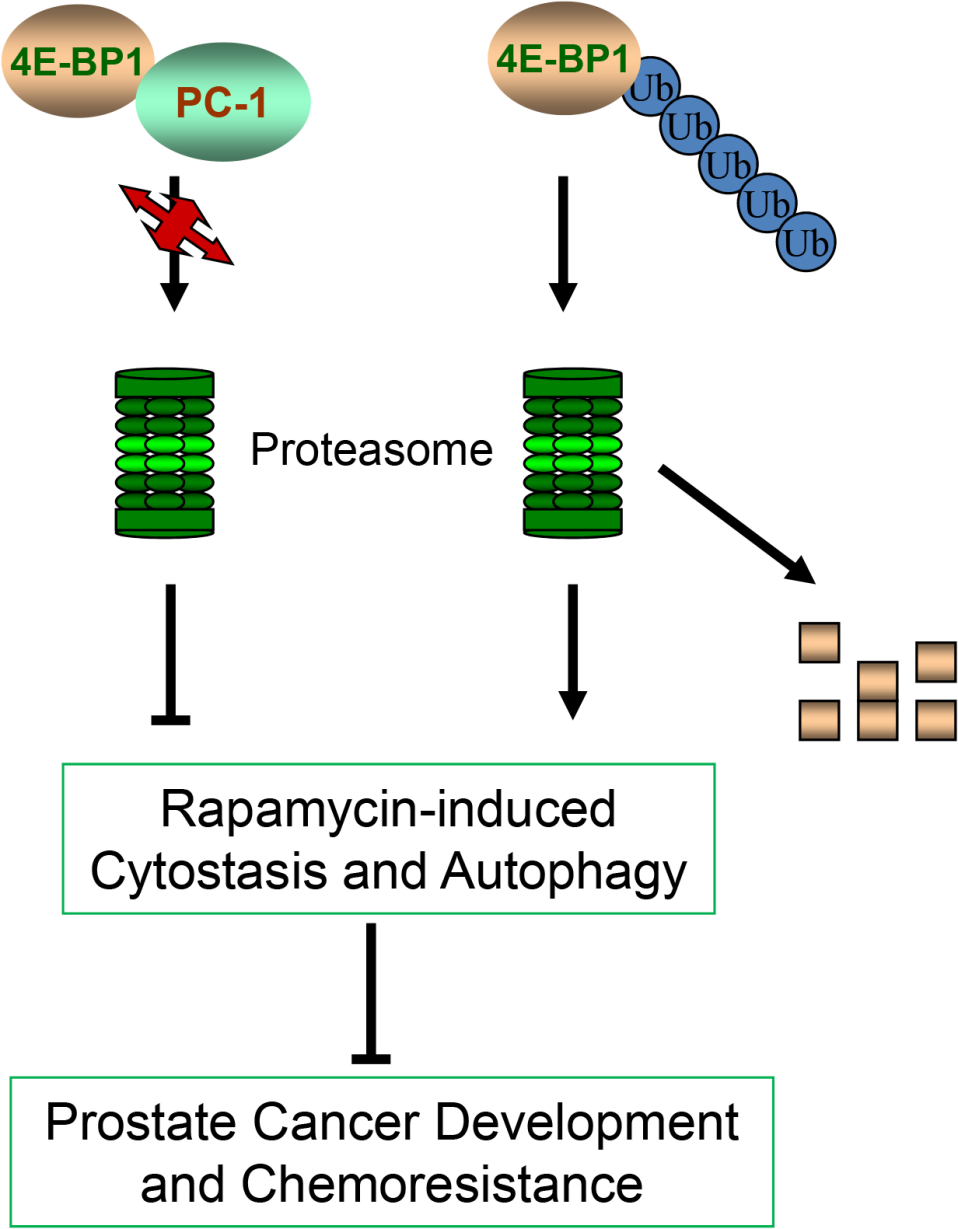
Model of molecular mechanism of 4E-BP1 degradation and the role of PC-1 in 4E-BP1 protection PC-1 prevented 4E-BP1 ubiquitination and proteasome-mediated degradation, antagonizing rapamycin-induced cytostasis and autophagy, promoting PCa progression and chemoresistance.

It is reported that specific cleavage of 4E-BP1 induced by activation of p53, which is 3 kDa smaller than the full-length protein and retains the C-terminal region of 4E-BP1 [50], is mediated by the proteasome and blocked by MG132 [51] through which 4E-BP1's stability was regulated. Recently, Yanagiya's group proposed that non-eIF4E-bound hypophosphorylated 4E-BP1 is prone to degradation in ubiquitination and proteasome pathway, which relies on KLHL25-CUL3 ubiquitin ligase. In this way, eIF4E activity and the translation homeostasis are tightly controlled [52, 53]. Here, we report that PC-1 regulates 4E-BP1 protein possibly by affecting stabilization of 4E-BP1 protein through the ubiquitin-proteasome pathway. Our data show that PC-1 interacts directly with 4E-BP1 and colocalizes to the cytoplasm and regulates 4E-BP1 protein *in vivo* and *in vitro*. Accordingly, PC-1 overexpression decreases the half-life of 4E-BP1 protein in LNCaP cells, but silencing of PC-1 increases protein half-life. MG132 blocks 4E-BP1 degradation in PC-1-depressed C4-2 sh cells and PC-1 overexpression decreases 4E-BP1 ubiquitination. Thus, PC-1 regulates the stabilization of 4E-BP1 protein by affecting its ubiquitination and degradation. In summary, our results demonstrated that expression of PC-1 confers sensitivity of PCa cells to chemotherapy, and decreases cell cycle arrest and autophagy induced by rapamycin. PC-1 stabilizes 4E-BP1 protein by inhibiting its ubiquitination and proteasomal degradation, which reveals another mechanism by which PC-1 enhances PCa cell survival and malignant progression (Fig. 7). Our findings provide new insight about the role of PC-1 and 4E-BP1 in cellular physiology and PCa progression and imply that PC-1-4E-BP1 interactions might be an excellent therapeutic target for advanced PCa.

## MATERIALS AND METHODS

### Cell lines and medium

Prostate cancer cell lines LNCaP and C4-2 were grown in RPMI 1640 (Invitrogen, Carlsbad, CA, USA) with 8% fetal bovine serum (FBS, Hyclone, Hudson, NH, USA), 10 mmol/l HEPES, and 1.0 mmol/l sodium bicarbonate and incubated at 37°C with 5% CO2 in a humidified chamber. LNCaP-zero and LNCaP-pc-1 were generated from LNCaP cells as described previously [27]. C4-2 NC and C4-2 sh generated from C4-2 cells, by stably transfecting with specific shRNA constructs targeting the PC-1 gene coding region (nucleotides 394~414, shPC-1) and a control construct (NC), respectively.

### Plasmids and antibodies

The sequence of PC-1 was amplified from human PC-1 cDNA and the PCR products were sub-cloned into pGEX-5T, pEGFP-N1, and pCMV-2B-tag vector, respectively. The sequence of 4E-BP1 was amplified from human 4E-BP1 cDNA and the PCR products were sub-cloned into pGEX-5T, pET22b, pEGFP-N1, and pDsRed-N1 vetor, respectively. The plasmid encoding Myc-ubiquitin was a gift from Dr. Cheng Cao (Beijing Institute of Biotechnology, Beijing, China). The specific shRNA constructs targeting the PC-1 and control construct was obtained from Cenechem Company (Shanghai, China). For immunoprecipitation and western blotting, the following primary antibodies were used: polyclonal antibody against PC-1 N-terminal 46 amino acids residues made by our laboratory [27], β-actin (Santa Cruz Biotechnology, Inc, Dallas, TX); antibodies against 4E-BP1, p4E-BP1(T70), mTOR, pmTOR(S2481), pmTOR(S2448), EIF4A, pRb(S780) and LC3B (Cell Signaling Technology, Danvers, MA); monoclonal antibodies against His (Invitrogen, Carlsbad, CA), Flag (Sigma-Aldrich, St Louis, MO), GFP (Abmart, Berkeley, NJ), MYC, Ub, p21, and p27 (Santa Cruz Biotechnology, Inc, Dallas, TX). The secondary antibodies were either horseradish peroxidase-linked anti-rabbit or anti-mouse both from Zhongshan Golden Bridge Biotechnology.

### Rapamycin sensitivity and colony-formation assays

Rapamycin was purchased from Merck Chemicals Ltd (Nottingham, UK) and used in LNCaP and C4-2 cells to determine cell sensitivity using 3-(4,5-dimethylthiazol-2-yl)-2,5-diphenyltetrazolium bromide (MTT) assay. Cells were seeded at a density of 2000-3000 per well in 96-well plates with 100 μl culture medium. Different concentrations of rapamycin were added in 100 μl culture medium the next day and untreated cells were controls. After 4 or 5 days, cell growth was measured [27]. Absorbance was normalized to controls. For the colony-formation assay, LNCaP and C4-2 cells were grown in 6-well plates in medium with rapamycin at 10 or 20 ng/ml, and this treatment was repeated every 2 days. After 15 to 20 days, the crystal violet experiment was performed.

### Immunoprecipitation and GST pull-down assays

LNCaP-pc-1 and LNCaP-zero lysates were immunoprecipitated to confirm the interaction between PC-1 and 4E-BP1 *in vivo*. Cells were lysed with lysis buffer [50 mmol/l Tris (pH 8.0); 50 mmol/l NaCl; 0.1% Nonidet P-40; protease inhibitor cocktail (Roche, Indianapolis, IN)] at 4°C for 45 min and centrifuged at 12,000 g for 10 min at 4°C. The supernatant was incubated with anti-myc antibody at 4°C for 3 h, and protein A/G-Sepharose beads (Santa Cruz Biotechnology, Inc, Dallas, TX) were added to the mixture and incubated for 6 h. Sepharose beads were washed with lysis buffer three times and resuspended in SDS-PAGE loading buffer for Western blot using anti-4E-BP1 antibodies. For the GST pull-down assay, Escherichia coli strain BL21 (DE3) transformed with pGEX-5T-PC1, pGEX-5T-4E-BP1, pET22b-4E-BP1 and the control vector pGEX-5T-1. After culture shaking to an OD600 of 0.6, isopropyl-h-d-thiogalactopyranoside was then added to a final concentration of 0.1 mM for 4 h. The His-4E-BP1 was purified as described (11). GST and GST-PC-1 and GST-4E-BP1 fusion protein was purified by GST-Sepharose beads (Pharmacia, Piscataway, NJ) according to the manufacturer's guidelines. 293T cells transfected with pCMV-2Btag-PC-1 lysates or purified His-4E-BP1 were incubated with immobilized GST or GST-fusion protein at 4°C for 3 h. Beads washed with lysis buffer 4 times and resuspended in SDS-PAGE loading buffer for Western blot using anti-His or anti-Flag antibodies.

### Immunofluorescence

293T, LNCaP, and C4-2 cells were seeded on slide covers in 6-well plates. Cells were transfected with the pEGFP-N1-PC-1 and pDsRed-N1-4E-BP1 vectors and fixed with 4% (v/v) paraformaldehyde after 48 h. Nuclei were stained with 100 μg/ml DAPI. Cells were viewed with a confocal laser scanning microscope.

### 4E-BP1 stability assay

To measure 4E-BP1 stability, LNCaP and C4-2 cells were treated with 40 μg/ml CHX and cells were harvested and lysed in lysis buffer at different time points (0, 4, 8, 12, and 24 h for LNCaP or 0, 4, 8, 12 and 24 h for C4-2). 4E-BP1 was measured with immunoblotting with anti-4E-BP1 antibody or β-actin as a control.

### Ubiquitylation assay

293T cells were single or co-transfected with 2.0 μg Myc-ubiquitin, 1.0 μg Flag-PC-1 and GFP-4E-BP1. After transfection (24 h), 10 μM MG132 was added to the cells for 18 h and cells were lysed as described above. Lysates were immunoprecipitated with anti-GFP antibody and analyzed by Western blot.

### Cell cycle analysis

LNCaP-zero and LNCaP-pc-1 cells were grown in 60 mm^2^ plates with/without 10 ng/ml rapamycin for 24 h. Cells were harvested and fixed with 70% ice-cold ethanol, followed by incubation in RNase A (100 μg/ml) at 37°C for 30 min. Cells then were stained with 40 μg/ml propidium iodide and analyzed by flow cytometry.

### Immunohistochemical analysis

Tissue microarrays of 40 prostate tumors were purchased from Shanxi Chaoying Biotechnology Co., Ltd. and the clinicopathologic features of all 40 patients were summarized in Supplementary Table 1. The microarrays were deparaffinized and hydrated in xylene and graded ethanol to distilled water. Antigen retrieval was performed with heating in retrieval buffer. Slides were incubated with primary antibody PC-1 (1:150) and 4E-BP1 (1:100) and stained in DAB solution (Zhongshan Golden Bridge Biotechnology Co., Ltd.) followed by incubation with Polink-2 plus kit PV-9001 (Golden Bridge International, Inc.), and counterstained with hematoxylin. Tissue arrays were scored manually using a 40× objective. Staining of cancer tissue received an intensity score of 1 for mild expression, 2 representing moderate expression, 3 representing high expression, and 4 representing highest expression (Supplementary Fig. 1). Tissue with intensity scores from 1 to 2 means low, whereas, the cancer tissue intensity scores from 3 to 4 means high. Statistical comparisons were made with SAS.

### Statistical analysis

Statistical calculations were performed using SPSS 13.0. The data in this study were presented as means ± standard deviation (sd). Student's *t* test and x2 test were used when appropriated. #*p* < 0.01, *p < 0.05 was considered significant.

## SUPPLEMENTARY FIGURE AND TABLE



## Abbreviations

4EBP1: eIF4E-binding protein 1
eIF4E: eukaryotic translation initiation factor 4E
p70S6K: ribosomal p70 S6 kinase
mTORC1: mammalian target of rapamycin complex 1
mTORC2: mammalian target of rapamycin complex 2
PI3K: phosphatidylinositol 3-kinase
