# Supplementary material for: PC-1/PrLZ confers resistance to rapamycin in prostate cancer cells through increased 4E-BP1 stability
Source: Oncotarget. 2015 May 11;6(24):20356–69. doi: 10.18632/oncotarget.3931 (PMC4653010; doi:10.18632/oncotarget.3931)
Supplement: Supplementary file 1 [file oncotarget-06-20356-s001.pdf]

SUPPLEMENTARY FIGURE AND TABLE

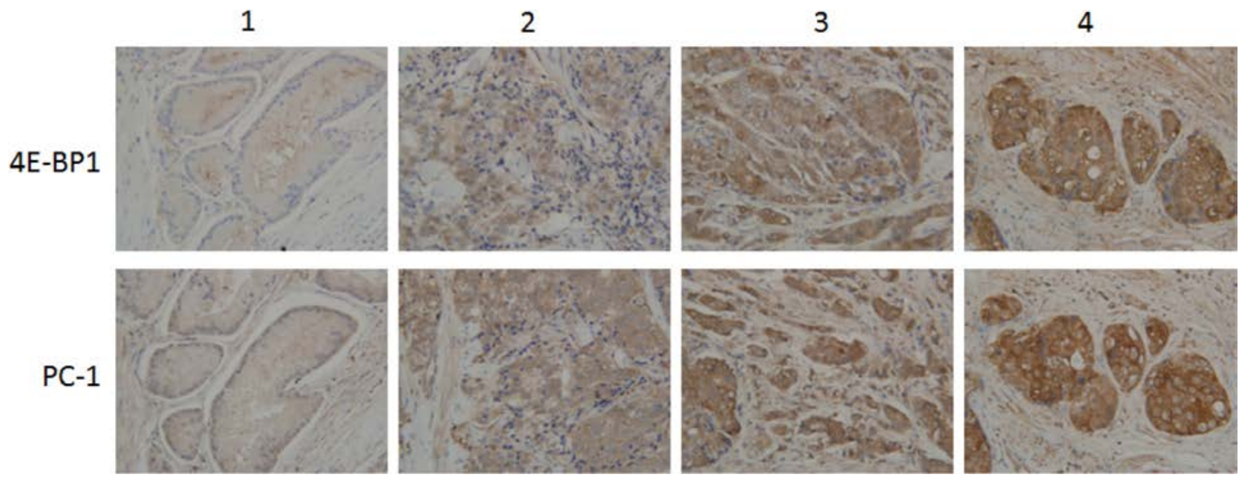

Supplementary Figure S1: Representative images of IHC staining illustrating the scoring of 4E-BP1 and PC-1 in PCa tissues.

Supplementary Table S1. Clinical features of 40 patients with prostate cancer (PCa)

Characteristics	n
**Age**	
≤mean (65)	14
>mean (65)	26
Range (20-87)	
**T stage**	
1-2	34
3-4	6
**TNM stage**	
I/II	20
III/IV	20
**Gleason**	
Gleason I	1
Gleason II	12
Gleason III	9
Gleason IV	12
Gleason V	6
